# The elusive cause of syncope: Left atrial myxoma with mitral valve obstruction

**DOI:** 10.1002/ccr3.7654

**Published:** 2023-07-17

**Authors:** Mahfujul Z. Haque, Muhammad Ahmed, Abdul Mannan, Syeda Reesha, Mashkur Husain

**Affiliations:** ^1^ Michigan State University College of Human Medicine Grand Rapids Michigan USA; ^2^ Wayne State University School of Medicine Detroit Michigan USA; ^3^ Department of Internal Medicine University of Buffalo Buffalo New York USA; ^4^ Interventional Cardiology of Downriver Heart and Vascular Specialists Southgate Michigan USA

**Keywords:** left atrial myxoma, left ventricle, neovascularized, pulmonary embolism, resection

## Abstract

**Key Clinical Message:**

Syncopal patients should be evaluated for cardiac causes, including myxoma, as highlighted in this case. Transthoracic echocardiography and coronary angiography are valuable diagnostic tools. Interventional cardiology plays a crucial role in the successful treatment of myxoma.

**Abstract:**

This case video describes the presentation and successful treatment of a 58‐year‐old woman who experienced recurrent episodes of syncope. After ruling out other health concerns, a pulmonary embolism was suspected and further investigations revealed a mass in the left atrium causing significant obstruction of the mitral valve. The mass, identified as a neovascularized myxoma, was successfully resected, emphasizing the significance of considering myxoma as a potential cause of syncope and highlighting the role of interventional cardiology in its management.

A 58‐year‐old woman presented with successive syncopal episodes with no other health concerns. Physical examination revealed no abnormalities. Pulmonary embolism was suspected, and CT revealed a mass. Transthoracic echocardiography demonstrated an echogenic mobile mass with stalk in the left atrium causing near‐complete mitral valve obstruction. The mass occupied approximately 75% of the atrial chamber with a left ventricular diastolic projection (Video S1). Coronary angiography revealed a highly neovascularized myxoma supplied by the left circumflex artery (Video S2). The patient underwent successful resection. Pathological findings were consistent with myxoma. This case highlights the importance of considering myxoma in syncopal patients and the role of interventional cardiology in its treatment.^1^

## AUTHOR CONTRIBUTIONS

**Mahfujul Z. Haque:** Conceptualization; data curation; formal analysis; investigation; methodology; resources; validation; visualization; writing – original draft; writing – review and editing. **Muhammad Ahmed:** Conceptualization; data curation; formal analysis; investigation; methodology; resources; software; validation; visualization; writing – original draft; writing – review and editing. **Abdul Mannan:** Conceptualization; data curation; formal analysis; investigation; methodology; resources; software; supervision; validation; visualization; writing – original draft; writing – review and editing. **Syeda Reesha:** Project administration; supervision; writing – original draft; writing – review and editing. **Mashkur Husain:** Project administration; resources; supervision; visualization; writing – original draft; writing – review and editing.

## FUNDING INFORMATION

None.

## CONFLICT OF INTEREST STATEMENT

None.

## CONSENT

Written informed consent was obtained from the patient to publish this report in accordance with the journal's patient consent policy.

## Supporting information


Video S1.

Video S2.

## Data Availability

The data that supports the findings of this study are available in the supplementary material of this article.
